# DeepFake knee osteoarthritis X-rays from generative adversarial neural networks deceive medical experts and offer augmentation potential to automatic classification

**DOI:** 10.1038/s41598-022-23081-4

**Published:** 2022-11-03

**Authors:** Fabi Prezja, Juha Paloneva, Ilkka Pölönen, Esko Niinimäki, Sami Äyrämö

**Affiliations:** 1 Faculty of Information Technology, University of Jyväskylä, 40014 Jyväskylä, Finland; 2 Department of Surgery, Central Finland Healthcare District, 40620 Jyväskylä, Finland; 3 School of Medicine, University of Eastern Finland, 70211 Kuopio, Finland

**Keywords:** Osteoarthritis, Computer science, Scientific data

## Abstract

Recent developments in deep learning have impacted medical science. However, new privacy issues and regulatory frameworks have hindered medical data sharing and collection. Deep learning is a very data-intensive process for which such regulatory limitations limit the potential for new breakthroughs and collaborations. However, generating medically accurate synthetic data can alleviate privacy issues and potentially augment deep learning pipelines. This study presents generative adversarial neural networks capable of generating realistic images of knee joint X-rays with varying osteoarthritis severity. We offer 320,000 synthetic (DeepFake) X-ray images from training with 5,556 real images. We validated our models regarding medical accuracy with 15 medical experts and for augmentation effects with an osteoarthritis severity classification task. We devised a survey of 30 real and 30 DeepFake images for medical experts. The result showed that on average, more DeepFakes were mistaken for real than the reverse. The result signified sufficient DeepFake realism for deceiving the medical experts. Finally, our DeepFakes improved classification accuracy in an osteoarthritis severity classification task with scarce real data and transfer learning. In addition, in the same classification task, we replaced all real training data with DeepFakes and suffered only a $3.79\%$ loss from baseline accuracy in classifying real osteoarthritis X-rays.

## Introduction

Over the past decade^1,2^ , the use of artificial intelligence in medicine has increased substantially. Alongside the big boom of deep machine learning methods^3^, medicine became an integrative field for artificial intelligence. Currently, deep learning in medicine pertains mainly to clinical decision support and data analysis. By analyzing medical data for underlying patterns and relationships, deep learning systems have a broad range of applications, ranging from patient outcomes prediction^4–6^, diagnostics and classification^7–10^, and data segmentation^11,12^to the generation^13–17^ and anonymization of datasets^18–21^ with synthetic medical data.

Policy and regulatory directives concerning medical data privacy and use continue to be updated globally. The US Health Insurance Portability and Accountability Act (HIPAA)^22^ is similar to the General Data Protection Regulation (GDPR)^23^; both were developed to restrict data flow and ascertain patient consent for health data dissemination. GDPR is the strictest policy^16,24^ concerning medical data and is implemented in addition to any EU national data policies. Such approaches further complicate implementation and downstream relevance to research groups. In the current regulatory landscape, anonymized medical data cannot be distributed between countries given the potential for re-identification of individuals. Re-identification was shown to be possible even with small combinations of anonymized variables^25–27^. Intercontinental health data exchange further complicates the issue. When health data is shared from an EU country to a third country, the third country must prove equivalent data protection mechanisms as in the GDPR^28^. Research data is practically impossible to share without prior preparation, formal agreements, and careful planning. However, deep learning applications require large open datasets and publicly available contributions^29^ to improve further.

Generating DeepFake data has been identified as a prominent solution to these privacy issues and regulatory restrictions^18–21^. High-quality DeepFake data generated by artificial neural networks may effectively retain relevant medical information for medical research and deep learning tasks^30^. DeepFake data can be open-sourced and shared freely between research groups and the broader public. This approach satisfies regulatory requirements and allows research groups to cooperate and improve deep learning solutions. Within research groups, DeepFake data can be mixed alongside real data in an additive augmentation approach. These approaches have shown promise in improving the performance of deep learning solutions in medicine^14,15,31–34^.

This study focused on osteoarthritis data; osteoarthritis (OA) is currently the fourth most common source of disability worldwide^35^, with estimated costs of up to 2.5% of national growth product in Western countries^36^. Clinically, the knee is the most common site of osteoarthritis^37^. Knee joint osteoarthritis (KOA) manifests with cartilage degeneration, narrowing of the joint space, and development of bony deformities. In addition, bone spurs (osteophytes) typically develop. The disease does not have a cure and typically may lead to surgery and chronic side effects. However, if diagnosed early, the clinical progression can potentially be slowed, and the quality of life and mobility of the patient may be improved. The early diagnosis of osteoarthritis presents a significant challenge to medical experts and artificial neural networks^37,38^. The main reason is the faint radiographic indicators of the disease’s onset in the early stages. The Kellgren and Lawrence (KL) osteoarthritis rating instructions^39^ are the most commonly used “top-down” classification system of patient X-rays into different developmental stages of osteoarthritis. The KL 0 grade indicates no radiologic presence of osteoarthritis; grade 4 indicates severe osteoarthritis (illustrated in Fig. 6). In deep learning, image features are learned in a “bottom-up” hierarchy from large osteoarthritis imaging datasets . The learned features are used with a classification algorithm to predict Kellgren and Lawrence grades^38^. As with any other deep learning approach in medicine, privacy and anonymization are important, while more data may be needed to improve current solutions.

Convolutional neural networks (CNNs)^40^ are essential in deep learning KOA research^38^. The main reason is that CNNs are a fundamental block in modern deep learning^3^ and have caused a significant performance explosion in object recognition, classification, segmentation, and clustering approaches. Along with these “classical” tasks, new applications emerged, such as neural style transfer^41^, super-resolution^42^ , and text-to-image generation^43^. A new type of neural network to which some of these applications owe their success is the generative adversarial neural network (GAN)^44^. These networks made it possible to generate synthetic (DeepFake) data given an adequate amount of real data. GANs for imaging typically employ convolutional blocks and involve two neural networks opposed to one another in a min-max game. One neural network generates DeepFake images to fool the other network tasked with classifying between DeepFake and real data. The simultaneous training of these neural networks can eventually produce a Nash equilibrium^45^.

In medicine, GAN-based synthesis can be seen in various data domains such as computer tomography scans^46–48^, X-ray images^49–51^ , and magnetic resonance imaging^52–55^ . Broadly, GANs are often used for anonymization and augmentation tasks in medicine^14,15,18–21,31–34^. In the first case, GANs completely replaced real data, while in the latter, they complemented real data by increasing the data size with DeepFake data. However, augmentation effects vary between medical contexts, and medical experts have validated only a few systems^14,15,49^.

This paper presents DeepFake X-ray images of different knee joint osteoarthritis severities. DeepFake imaging data have been under development recently with noteworthy successes^14,15,31–34^. To the best of our knowledge, no such attempts have been made in osteoarthritis. The current best X-ray KL multi-class classification accuracy stands at 74.81%^38,56^. However, no previous method employed privacy-preserving data nor additive augmentation with DeepFake images. In this study, we developed two generative adversarial neural networks that can produce an unlimited number of knee osteoarthritis X-rays at different Kellgren and Lawrence stages. First, we validated our system with 15 medical experts, then showed anonymity and augmentation effects in deep learning. The resulting DeepFake X-ray images can be published openly and distributed freely among scientists and the general public.

## Results

We trained two Wasserstein generative adversarial neural networks with gradient penalty (WGAN-GP)^57^. We trained to produce nearly anatomically accurate X-rays of knee joint osteoarthritis. We assessed the extent of overall realism with a medical expert survey. In addition, we validated these generative models to augment and completely substitute the training data in a KL classification task performed by another neural network. In the first section of the results, we visualize the GAN training results. The second part relates to the medical expert survey. The third and final part presents results on anonymization and training augmentation.

Figure 7 shows our neural network design result, which consisted of two blocks. The generator block was built primarily with upsampling and 2D convolution modules with exponential unit activations and batch normalization. The same philosophy was followed for the discriminator block but excluding the upsampling and the batch normalization modules. The discriminator module was distinct because of the dropout layers to combat overfitting. The two blocks had a similar number of parameters, 6,304,900 for the generator and 6,335,861 for the discriminator; it would have been hard for either block to have an advantage while training. The generative network was trained twice independently, each time for 1000 epochs- the first time with classes KL 0 and 1 (none to doubtful OA) and the second time with classes KL 2, 3 and 4 (mild to severe OA). We used an exponentially decaying learning rate; as training progressed, changes to DeepFake images would be reduced to minor fine-tuning.

**Figure 1:**
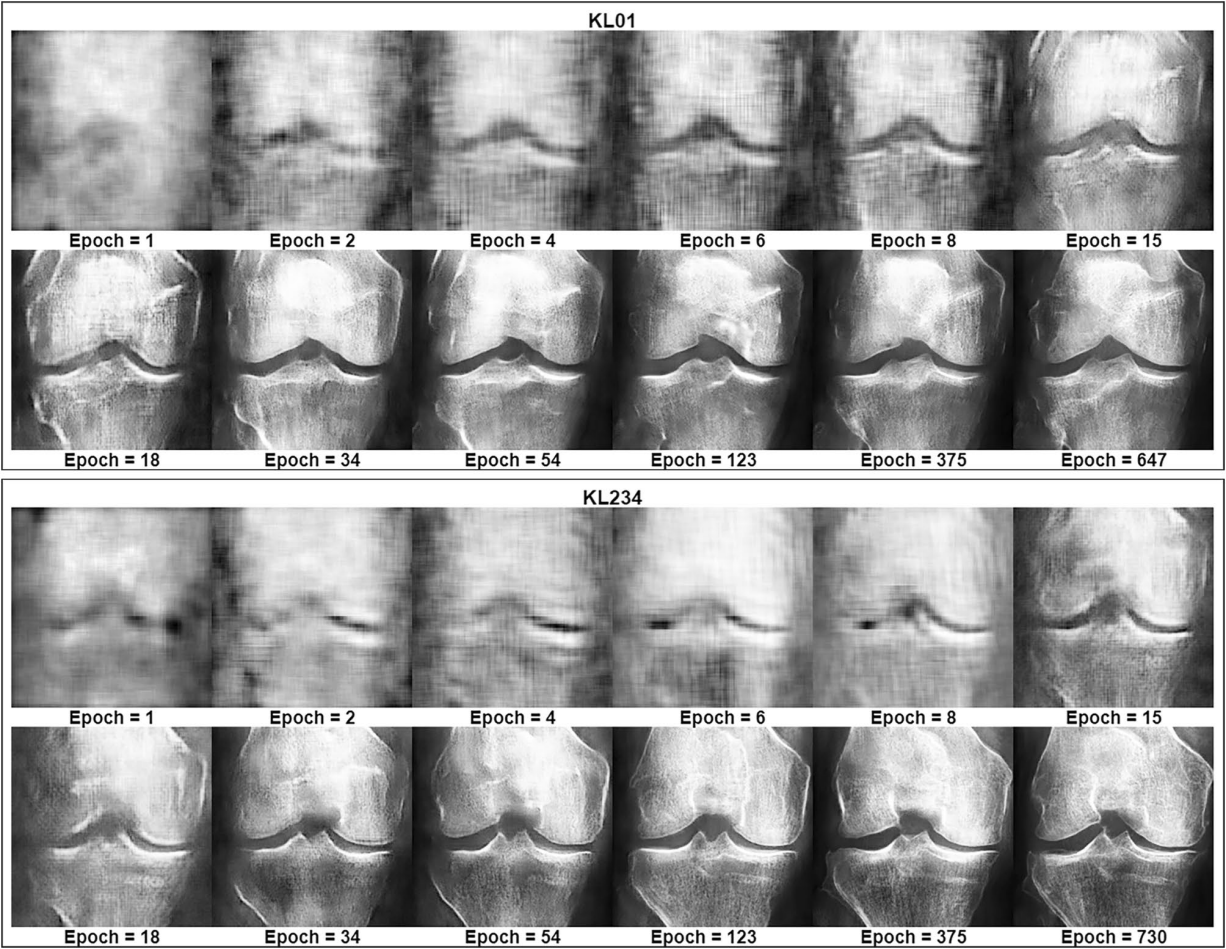
Training progress visualization with fixed latent space representation. Training improvements occurring over time are depicted for the KL01 and KL234 WGANs. All images in this figure are DeepFake.

Figure 1 shows epoch monitoring results with fixed latent space coordinates. The images spanned from early training up to the best-selected models for KL01 WGAN and KL234 WGAN. We identified a clear pattern of improvement in terms of overall anatomy, X-ray texture, and contrast conditions. The figure contains post-training fixed latent space coordinates entering the generator at different saved epochs from the KL01 and KL234 WGAN training; We observed that major structural changes began to diminish as the training progressed while texture changes continued to improve. For example, in the KL01 model, we observed a particular focus on structural features, such as the overall shape of the knee joint. After epoch 18, we observed a focus on texture changes as the shape of the patella became more pronounced. We saw similar patterns of improvement in the KL234 model. Contrary to KL01, the KL234 knee joint shape became less smooth and more sharp-edged, as is common in advanced stages (Fig. 6) of osteoarthritis. Finally, we observed excessive white regions indicating sclerosis were reduced after epoch 18.

### DeepFake realism survey

**Figure 2:**
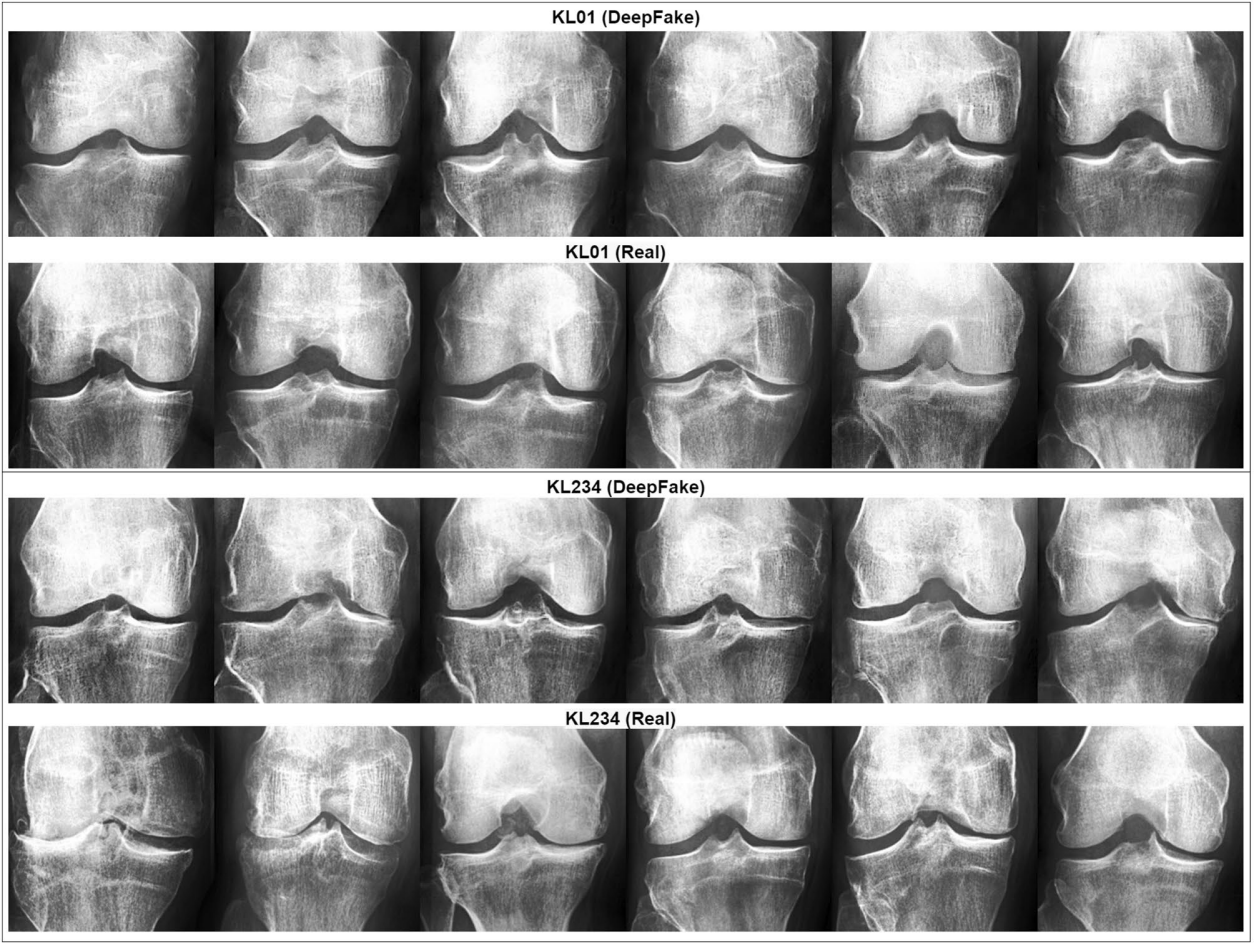
A sample from the survey images shown to medical experts. The top half includes KL01 DeepFake and real images, and the bottom half KL234 DeepFake and real images.

In order to assess the quality and medical accuracy of the generated images, we surveyed ten specialists in radiology and five specialists in orthopedic surgery. The medical experts practiced in the Hospital Nova of Central Finland Healthcare District , Finland. We presented 30 real and 30 DeepFake images from both the KL01 and KL234 classes, randomly selected and in random order. The task was to identify whether an image was authentic or synthetic. In addition, medical experts were asked to rate both real and synthetic images with respect to OA severity. The experts were not told how many of the 60 images were DeepFake. Figure 2 showcases 12 real and 12 DeepFake examples used in the survey. The DeepFake images were randomly generated from the best selected generative models. Figure 6 can assist the interpretation of Fig. 2.

Table 1 shows the scores of medical experts who classified the images as either DeepFake or real. We found that the average accuracy achieved amongst medical experts was 61.35%. The orthopedic surgeons alone achieved 65.25%, followed by the radiologists with 59.40%. However, there were fewer orthopedic surgeons who took the survey than radiologists.

**Table 1:** Medical expert accuracy, precision, and F1 score in classifying images as real or DeepFake, with standard deviations shown in parentheses. F1 score refers to the harmonic mean of the precision and recall metrics.

Medical experts	Accuracy	Precision (%)	F1 score (%)
Orthopedic surgeons $(n=5)$	65.25% (± 6.95%)	65.81	65.09
Radiologists $(n=10)$	59.40% (± 12.01%)	59.95	58.96
All	61.35% (± 10.71%)	61.91	61

We decomposed binary class average accuracy on a per-class basis in Table 2. We found 59.89% for DeepFake images and 62.81% for real images. For radiologists, we observed 56.17% for DeepFake images and 62.63% for real images. In contrast, orthopedic surgeons achieved 67.33% accuracy for DeepFake images and 63.17% for real images. These results suggested that, on average, DeepFake images were at least equally confusing to experts as were the real images. This finding indicated that the realism in the fake images was sufficiently high to deceive the medical experts. The result showed that more fake images were mistaken for real than the reverse.

**Table 2:** Single-class accuracy and F1 score for all and each medical expert group, with standard deviations shown in parentheses.

Medical expert	Accuracy (DeepFakes)	Accuracy (Real)	F1 score (DeepFakes) (%)	F1 score (Real) (%)
Orthopedic surgeons $(n=5)$	67.33% (± 3%)	63.17% (± 7.48%)	65.67	64.51
Radiologists $(n=10)$	56.17% (± 6.67%)	62.63% (± 5.11%)	57.18	60.74
All	59.89% (± 6.02%)	62.81% (± 2.76%)	60.01	61.99

**Figure 3:**
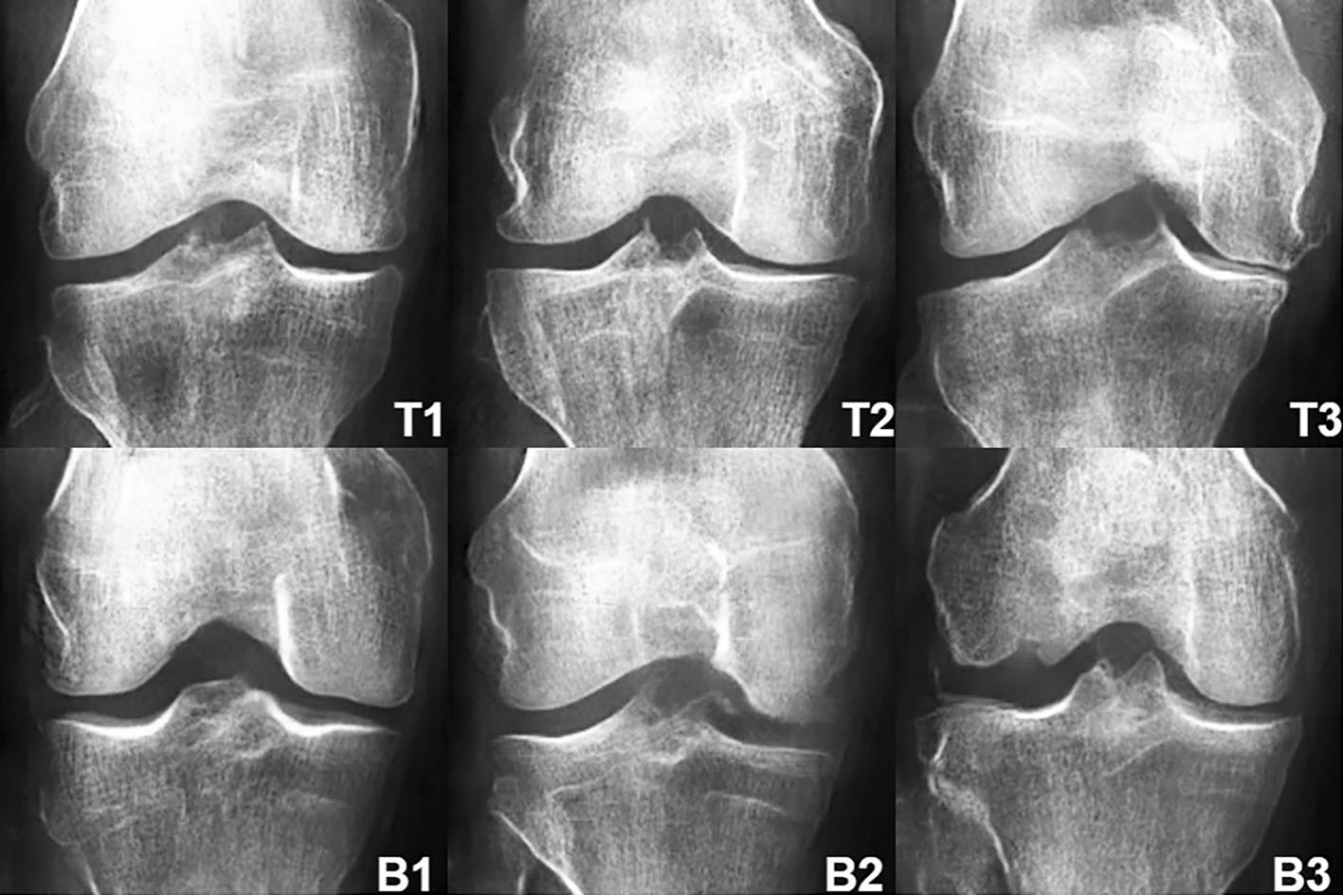
DeepFake top and bottom examples as a function of experts confused, T1 confused 85.71% of experts, T2 73.33%, T3 71.43%, B1 0%, B2 and B3, 13.33% each. Refer to Fig. 6 for interpretation assistance of the KL criteria amongst these images.

We expected that DeepFake images might confuse the experts to varying degrees. On a per-image analysis, Fig. 3 shows the three most misclassified (classified as real) DeepFake images ($>70\%$ of experts) and the three least misclassified ($<13\%$ of experts). Within the least misclassified, the bottom 1 (coded B1) had no experts confused. The latter results were not surprising, given that our GAN could occasionally exceed anatomical constraints and produce slightly or markedly exaggerated structural features. This analysis was essential since average accuracy may be elusive in describing such inter-sample variance.

**Table 3:** Average agreement between our medical expert labels, the original, and DeepFake labels. The agreement score is the average accuracy of all individual accuracy scores per medical expert. The standard deviation is shown in parentheses.

Medical expert	Rating agreement (DeepFake KL01)	Rating agreement (Real KL01)	Rating agreement (DeepFake KL234)	Rating agreement (Real KL234)
Orthopedic surgeons $(n=3)$	68.89% (± 40.76%)	88.89% (± 20.57%)	68.89% (± 38.76%)	55.56% (± 39.17%)
Radiologists $(n=9)$	88.43% (± 17.66%)	90.28% (±13.86%)	54.07% (± 31.95%)	51.85% (± 36.04%)
All	83.48% (± 21.62%)	89.44% (±13.50%)	57.78% (± 32.65%)	52.78% (± 35.45%)

Table 3 shows that all experts rated 83.48% of the DeepFake KL01 class correctly, similar to real KL01 with 89.44% accuracy. For the DeepFake KL234 class, the experts rated 57.78% of the items in agreement with the original labels. However, the experts rated the real KL234 with only 52.78% agreement against the original labels. We observed extensive standard deviations caused by extreme fluctuations in expert scores. Large intra-rater variance was present with both DeepFake and real images.

### Training augmentation and anonymization

Table 4 shows losses and accuracy for the validation and testing sets for each dataset (with and without DeepFake data augmentation), including the anonymized dataset. Specifications for transfer learning and datasets design are shown in Tables 5 and 6. The binary classification task predicted between KL01 and KL234 OA severities. We saw that losses were lower for the DeepFake augmentation set and validation accuracy followed upwards. All accuracy scores and losses were better in the augmentation sets at testing time. The most potent augmentation effect appeared at +200% Fakes with a testing score of 75.76%. We observed that the best validation accuracy was nearest to testing accuracy. This effect indicated better augmentation than the other entries. Notably, accuracy slightly decreased when we replaced all training data with DeepFake data. Testing accuracy decreased by $3.79\%$ compared to the baseline real data set. This minor accuracy drop indicated that the data remained OA-grade informative and anonymized. Overall, these augmentation and anonymization effects signaled a potential for positive downstream effects in knee osteoarthritis classification.

**Table 4:** Accuracy scores and losses for each dataset used for augmentation and anonymization.

Dataset	Testing accuracy (@Best validation loss) (%)	Testing loss (@Best validation loss)	Validation accuracy (Best) (%)	Validation loss (Best)
Real	71.21	4.142	80.3	4.07
Real +50% Fakes	73.48	3.819	81.06	3.809
Real +100% Fakes	72.73	3.404	81.06	3.428
Real +150% Fakes	73.48	3.205	78.03	3.295
Real +200% Fakes	75.76	2.833	78.79	2.925
Replace Real 100%	67.42	4.280	78.45	4.301

**Table 5:** Augmentation and anonymity data sets.

Dataset	Training set image count	Validation set image count	Testing set image count
Real	200	132	132
Real +50% Fakes	300	132	132
Real +100% Fakes	400	132	132
Real +150% Fakes	500	132	132
Real +200% Fakes	600	132	132
Replace Real 100%	200	132	132

**Table 6:** VGG16 transfer learning variant.

Layer type	Shape	Number of parameters
VGG16 (without output layer)	$6 \times 6 \times 512$	14,714,688
Flatten layer	18,432	0
Dense layer + ELU	256	4,718,848
Output layer + sigmoid	1	257
Total parameters: 19,433,793		
Trainable parameters: 11,798,529		
Non-trainable parameters: 7,635,264		

### Latent dimension exploration

**Figure 4:**
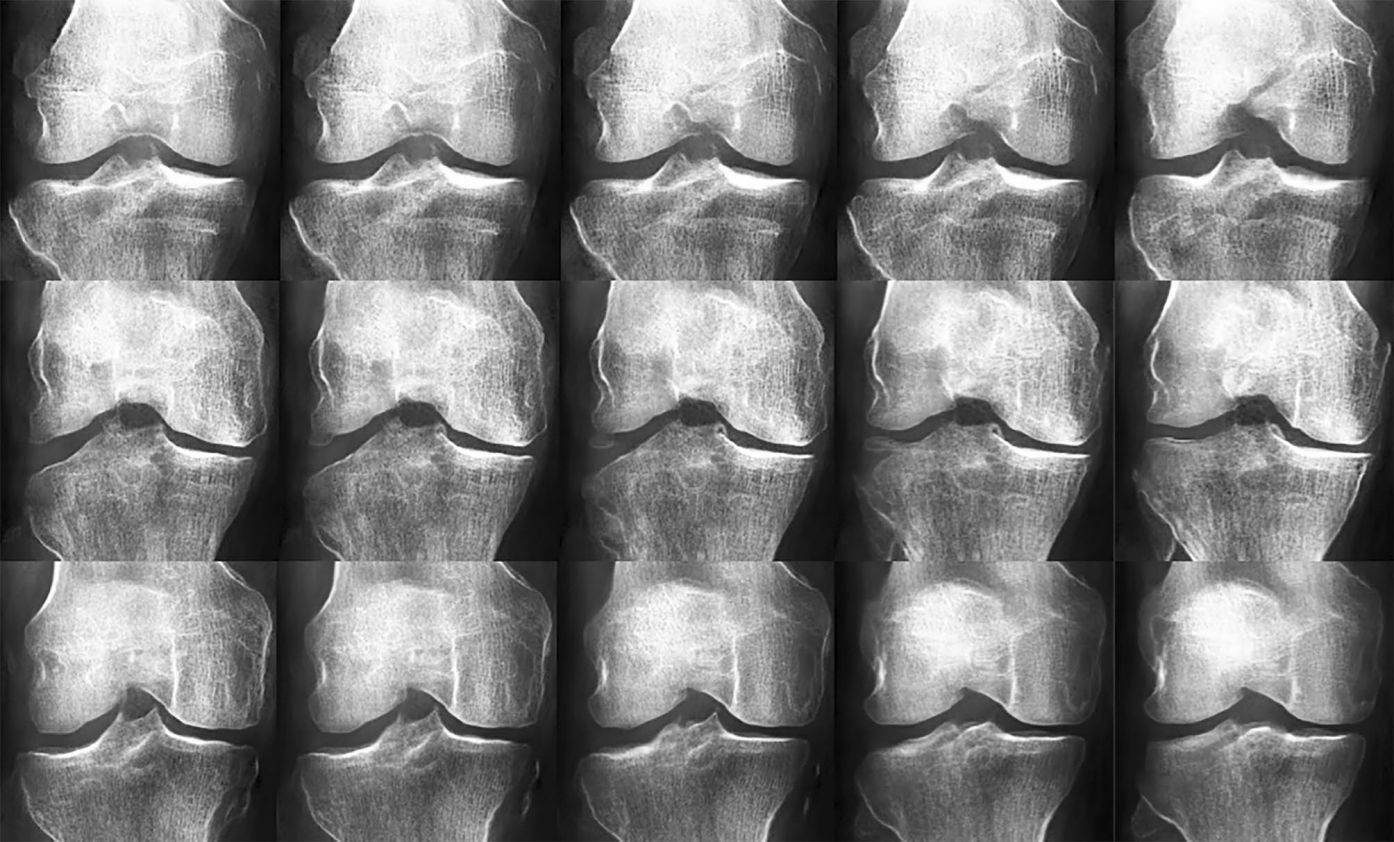
Latent dimension perturbations are displayed. The first two rows show changes in one latent dimension. Left to right for positive change, and vice versa for negative change. The last row showcases linear interpolation between two X-ray latent space coordinates at the extremes of the row.

GANs can visualize learned latent space representations; each latent dimension value change affects the resulting DeepFake. Our models were trained with 50 features/latent dimensions. Latent dimensions tend to be entangled after training and subsets may control one or more general high-level features. We generated three simple examples demonstrating future potential in designing KOA X-ray images. In the first two rows of Fig. 4, we show incremental changes in one latent dimension, from $-4.2$ to $+4.2$. The first row in the figure showed a random KL01 example, while the second row was a KL234 example. Ultimately, the last row showcases linear interpolation of all latent dimensions between two randomly generated X-rays at the row’s extremes. Upon closer view, we mainly observed changes to the intercondylar notch and lateral tibial condyle shape in the first row. In the second row, we observed that the lateral tibial condyle extended closer to the lateral femoral condyle. In that respect, the second knee joint image gradually became more symmetric. In the last row, we saw that the middle X-ray carried similar characteristics from both X-rays at the extremes of the row.

## Discussion

Our study demonstrated that generative deep neural networks could effectively generate medically realistic knee joint osteoarthritis X-ray images. This study introduced and validated such a system in computer vision osteoarthritis research and was the first to obtain related augmentation effects and anonymity by replacement. We showed that DeepFake knee-joint osteoarthritis X-rays retained relevant osteoarthritis and anatomical information. As a result, we rendered anonymization by replacement possible without substantial accuracy loss in deep learning. We showed that, on average, even medical experts had difficulties differentiating between our DeepFake and real data. In addition, we demonstrated a positive potential for additive augmentation. In data-scarce transfer learning, adding DeepFake images to real training data improved classification accuracy in detecting knee joint osteoarthritis severity. Such transfer learning approaches are common in medicine, where data are often scarce and hard to obtain. Finally, we highlighted the potential educational use of this system by modifying generated osteoarthritis X-rays to specifications. This approach could enable future interactive medical education and stress testing of deep learning systems.

Regarding GAN selection, WGAN-GP produced the first results for this medical context. WGANs were chosen for being long well-understood, effective, and relatively lightweight baselines. Although outside the scope of this study, training and validation with more advanced GAN objectives and different architectures could produce improved results. However, such an approach would require multiple expert validation surveys, which can be challenging to obtain. We believe our neural network architecture would be a good starting point for such future work.

Regarding GAN training, we trained independent unconstrained KL models in order of severity; therefore , the data size available as a whole was of paramount importance. We merged KL classes and obtained a larger pool of images for two models of osteoarthritis severities (KL01 and KL234). The combination of KL grades led to less label noise among early KL grades, Which is further discussed in the next paragraph. All images were laterally flipped in the same orientation. This step was necessary because we observed that the generative process would occasionally generate two fibulas or mix the orientation of other morphological components. We contrast-equalized the data to make morphological details more pronounced; while prototyping, we observed faster improvement on high-contrast images. Finally, we used focus filtering^58,59^ because we observed that wide gaps in X-ray focus and texture clarity would confuse the generator and lead to focused and unfocused textures being generated into the same image. We removed X-rays with surgery prosthetics and other visible distortions ( tearing and scratches) to minimize potential training interference. A 210 $\times$ 210 image size was used to avoid GPU memory overflow. We used the Frechet Inception Distance^60^(FID) metric for model selection. Selecting an epoch/model with this approach was prone to noise artifacts because the FID expected much larger data sizes for precise estimates. The FID cannot be estimated reliably for individual DeepFake images. Overfitting can be challenging to detect without manual sample inspection^61^. In this respect, we enhanced model selection with an orthopedic surgeon. The surgeon analyzed the nearest real image neighbors of DeepFake images. This approach was essential for ensuring privacy before replacing training data. In this regard, we validated that the generator was not replicating training examples. The approach is fully detailed in Methods, and the neighbor pairs are included under Data Availability.

Regarding the medical realism survey, we observed that experts had more difficulty classifying DeepFake images as DeepFake than real images as real. In addition, we saw similar KL rating agreements to real and DeepFake data labels. These results highlighted sufficiently high realism in DeepFake images. However, we also observed that the degree of realism varied between images, as shown in Fig. 3, which displays rankings of individual images versus expert misclassification rates. This effect was also present in the high standard deviations shown in the KL rating agreement task. To the best of our knowledge and according to the literature review, we had one of the largest medical expert samples. This proved essential to highlight the large variance in this validation task. As shown in Table 3, experts strongly disagreed with real and DeepFake KL234 labels. This phenomenon was not surprising, given that inter-rater variance exists and class KL 2 is frequently confused with KL 1 also in clinical settings. Similar results were found in the top^56^ deep learning solution for KL grading. The confusion matrices in that study showed that KL 1 and KL 2 were the leading cause for the average score to decrease. These outcomes aligned with clinical practice, where these particular confusions are also expected. Overall, some images had better clinical features than others for skewing the opinions of the medical experts. It is worth noting that we did not purposefully truncate the input noise distribution to the generator; although this would have led to relatively more stable DeepFake images, it would have decreased the diversity of DeepFake images. A limitation of the expert responses was that many participants completed the survey on portable devices (e.g., tablets, phones). Such devices typically have an excellent capacity to represent small-size images; while zooming was not prohibited or disabled during our survey, the choice of display device could have influenced the survey results. Due to GPU memory constraints, we generated images smaller than the typical resolution of X-ray images routinely used by medical experts. Overall, image size and resolution could influence the results ; thus, the study’s results only apply within those parameters. However, the image size generated was close to typical deep learning image sizes (299 $\times$ 299 pixels). Finally, the variance shown in Fig. 3 would introduce unwanted noise to applications such as landmark detection. Although it is outside the scope of this study, further integration of landmark labels could benefit generation and landmark detection. However, this approach would require expert landmark labels, which may be challenging to obtain.

Regarding the medical realism of GANs, we did not find GAN validations with medical experts other than in three studies^14,15,49^. Our study presented an equally large expert sample size as the largest found in the literature review^14^. DeepFake realism was elusive without external validation, especially for non-experts. Using metrics such as FID can be helpful, but cannot account for individual examples and small sample sizes. In this regard, seemingly accurate DeepFakes to developers might be flagged as inconsistent by medical experts or vice versa. Secondarily, visual features relevant to medical experts could differ from the neural network features used to calculate the FID. We strongly recommend collaboration between developers and medical experts during development and through validation surveys. We believe this approach could help complement current computational approaches, validate, and improve medical realism outcomes.

Regarding anonymization and augmentation, we completely replaced real training data with DeepFake with only a $3.79\%$ loss of testing accuracy on real data compared to the baseline. This result strongly indicated that anonymization by replacement was possible and that privacy concerns could be answered in this way effectively. To this end, the validation step between real and synthetic nearest neighbors was an effective way to investigate whether our WGANs replicated training images. The opposite effect would have caused privacy issues in anonymization with replacement. We suspected that the loss in accuracy could be due to DeepFake images being more focused than testing images . We tested with images derived from rejected GAN training images. Conversely, in augmentation, we observed that a positive trend in boosting testing accuracy existed as we increased the DeepFake data in the training set. Such data-scarce scenarios are common in the field of medicine, where data is either small or unavailable due to privacy policies and restrictions. Limitation-wise, the rejected real data (used as the scarce data source) and the fake data differed in texture quality and overall focus. These limitations could negatively impact obtaining more potent augmentation effects. In addition, the binary class set-up offered limited insight into augmentation effects for each sub-class. Nonetheless, the current results were promising. The augmentation effects aligned with similar effects found in other GAN-based augmentation studies^14,15,31–34^. We believe our neural network design could be adapted to achieve results with other radiologic data . More advanced vision systems (e.g., Inception^62^, Transformers^63^) could offer better classification accuracy. In this study, we chose a long well understood, common transfer baseline (VGG16^64^). It was outside the scope of this study to maximize accuracy in the augmentation task. The task only aimed to highlight the DeepFakes’ augmentation potential. However, a future study could investigate augmentation effects with multiple classifiers.

We demonstrated that our neural networks contained the necessary capacity to produce realistic DeepFake KOA images. What helped produce the given quality of fake images , other than the WGAN-GP objective, was the design of the architecture, the total number of parameters , and the relative similarity of architectures between the generator and discriminator. Implementing batch normalization, dropout regularization, and ELU activations along our architecture design showed significant potential in the development stages. We also believe that grayscale single-channel image inputs with small and decaying learning rates played a role. Limited real data naturally allowed for limited DeepFake structural variation ; DeepFakes mainly varied in line with the real data. In this regard, it was expected that the generator would suffer from sampling regions that produced limited and, at times, structurally questionable results. Such examples may be observed in the large DeepFake dataset we provided; some outliers can be found. Working with the FID metric with our sample size proved to be challenging, as the FID requires large data sizes to be more accurate. Thus , the relative value of the FID was informative, but the absolute values would have been affected. Evaluating minimum FID models with an external medical expert proved essential. We analyzed whether memorization (overfitting) occurred by comparing DeepFakes to their closest real nearest neighbors^65^ embeddings. It is essential to highlight that FID cannot help judge individual images, which limits the evaluation of individual images.

Finally, concerning latent space exploration, we saw a few examples of in-place editing of DeepFakes. Although outside the scope of this study, a thorough investigation of learned latent features might reveal several high-level features of clinical relevance. A future approach could focus on deriving a bidirectional GAN variant of the current system. One potential latent feature to be discovered could be the patient’s age. The age feature has appeared in other GAN implementations^66^. In osteoarthritis, age can play a catalytic role in disease progression. We speculate that the age feature might be entangled with a potential osteoarthritis grade feature. Ultimately, the ability to generate osteoarthritis’ state forward in time will be of immense prognostic value.

## Methods

**Figure 5:**
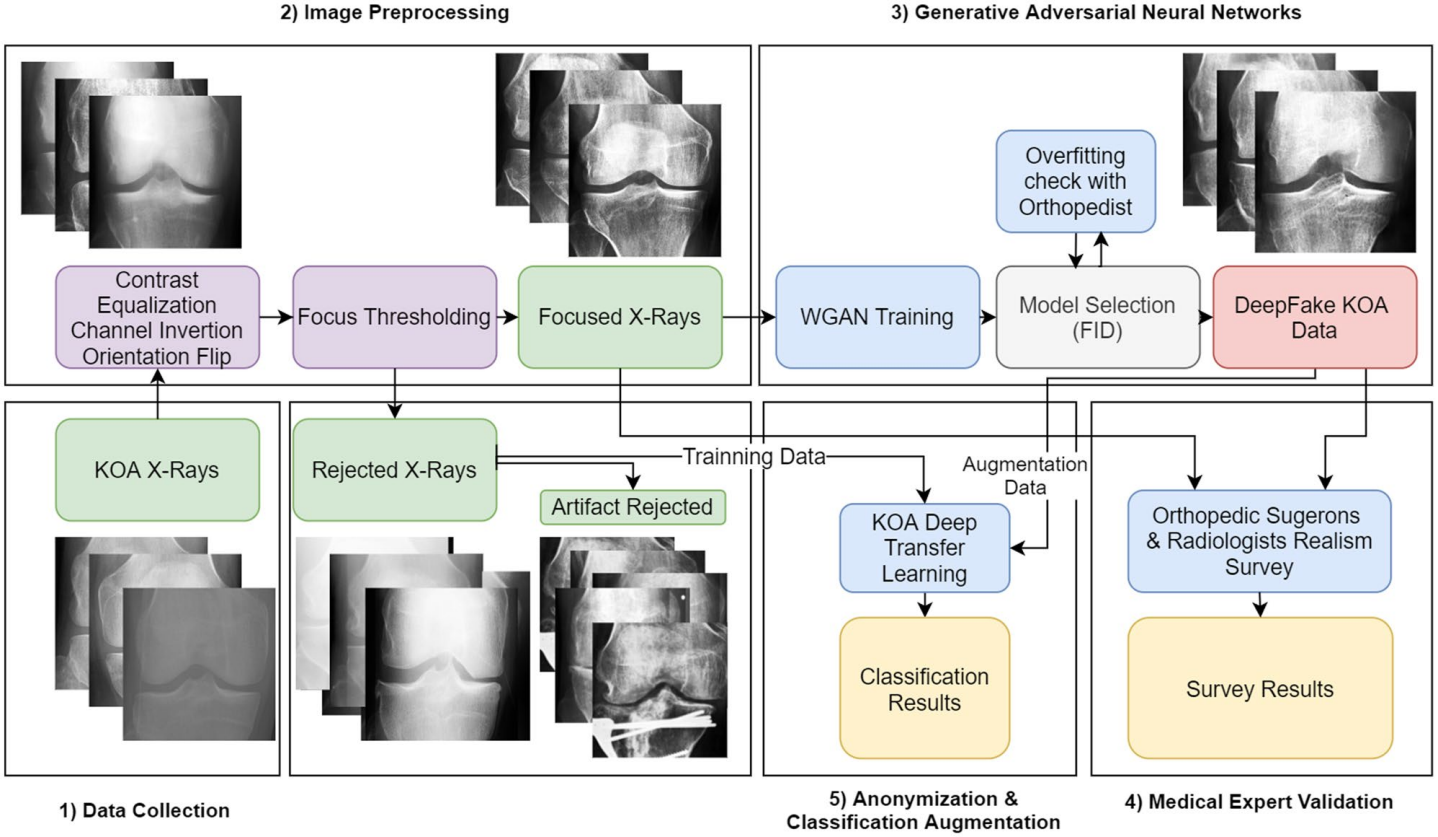
Flowchart of tasks and data involved in this paper. Real data are highlighted in green; DeepFake data are in red. Purple signifies data processing operations, and blue indicates classification-related procedures. Block headers contain ascending numbers to signify the order of operations (from 1 to 5).

Methods were divided into four sections. The first section dealt with data collection. The second section pertained to data processing, such as contrast equalization, channel inversion , and focus filtering. The third section dealt with generative adversarial neural network training and validation. The last section pertained to aggregating the results from the medical expert survey and transfer learning classification experiments. Figure 5 illustrates all parts in small comprehensive steps.

### Data collection

**Figure 6:**
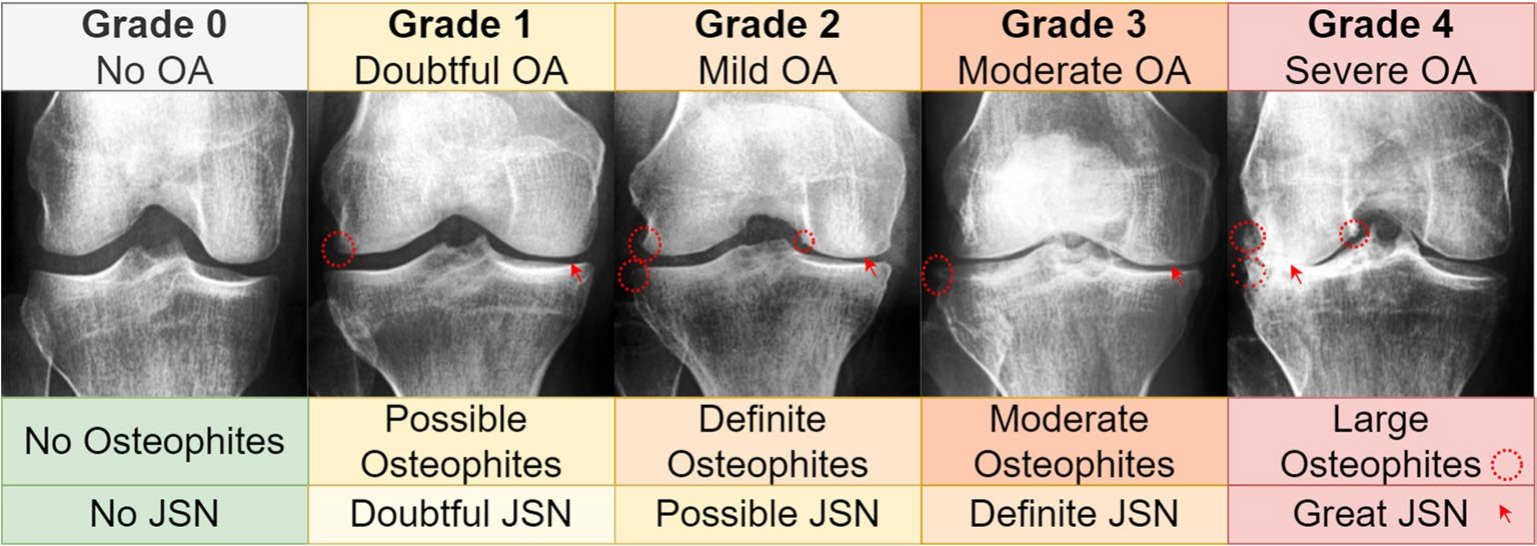
Random examples for each KL grade with main KL criteria. From left to right , we increase KL grade from 0 (no radiological signs of OA) to 4 (severe OA), JSN refers to joint space narrowing. Examples contain two red markers; the circular marker indicates regions with osteophytes. The arrow shows joint space narrowing.

We obtained knee joint X-ray images used by Chen 2019^67^, which processed data from the Osteoarthritis Initiative (OAI)^68^. The OAI data was were derived from a longitudinal multi-center effort to collect relevant biomarkers for identifying knee osteoarthritis onset and progression. The OAI study included 4796 participants with ages between 45 and 79 years. Our study used the pre-processed Chen 2019^67^ primary cohort data^69^, which employed automatic knee joint detection, bounding, and zoom level standardization to 0.14mm/pixel. The data contained 8260 individual knee joint images with a uniform size of 299 $\times$ 299 pixels. The images were derived from 4130 X-rays containing both knee joints and were graded with the Kellgren and Lawrence system^39^. Figure 6 shows a single image per osteoarthritis grade from the data. The distribution of images between KL grades was as follows: 3253 for grade 0, 1495 for grade 1, 2175 for grade 2, 1086 for grade 3, and 251 for grade 4. We merged KL 0 and KL 1 images into the KL01 class. Accordingly, KL 2, KL 3, and KL 4 were merged into the KL234 class. The number of images per KL grade was inadequate for training each grade separately. Dividing between KL01 (no to doubtful OA) and KL234 (mild to severe OA) is also relevant in clinical practice. Finally, class KL01 contained 4748 images, while the remaining 3512 images were included in the KL234 class.

### Image pre-processing

#### Rotation and histogram equalization

After merging KL levels, we laterally flipped all right-orientated images to the left. We detected and inverted negative channel images, of which we found 112 for KL01 and 77 for KL234. Next, we contrast-equalized the histograms of the images. We achieved this with Eq. (1), where for a given gray-scale image ${\varvec{{I}}}$ of $m\times n$ dimensions with cumulative distribution function *cdf* and pixel value *v* , we obtained an equalized value *h*(*v*) in the range [0, 255] by:

1 $$\begin{aligned} h(v)=255\frac{cdf(v)-cdf_{min}}{(m\times n)-cdf_{min}} \end{aligned}$$

where $cdf_{min}$ is a non-zero minimum value of the image’s cumulative distribution and $m\times n$ is the total number of pixels. Lastly, all images were re-scaled from $299\times 299$ to $210\times 210$ pixels. All steps were completed with the scikit- image^70^ and NumPy^71^ Python libraries.

#### Focus filtering

After contrast equalization, we aimed to separate X-rays concerning the image focus related to the overall blurriness of each image and texture clarity. To obtain this result, we used the Laplace variance threshold approach^58,59^. We first obtained the Laplacian of the image, which is the second derivative of the image and often used for edge detection. Considering an arbitrary grayscale image ${\varvec{{I}}}$ of size $m\times n$, the Laplacian was approximated by the following kernel (Eq. 2):

2 $$\begin{aligned} {\varvec{{L}}}= \begin{pmatrix} 0 &{}\quad -1 &{}\quad 0\\ -1 &{}\quad 4 &{}\quad -1\\ 0 &{}\quad -1 &{}\quad 0 \end{pmatrix} \end{aligned}$$

In this case $S({\varvec{{I}}})$ is the convolution of image ${\varvec{{I}}}$ with the Laplacian kernel ${\varvec{{L}}}$ with the resulting size of $m\times n$. Next (Eq. 3), the final focus metric was calculated as the variance of the absolute values for the convolved image.

3 $$\begin{aligned} S({\varvec{{I}}})_{var}=\sum _{i=1}^{m}\sum _{j=1}^{n}\left[ | S({\varvec{{I}}})_{i,j}|- S({\varvec{{I}}})_\mu \right] ^2 \end{aligned}$$

where $S({\varvec{{I}}})_\mu$ was the mean of values given by (Eq. 4):

4 $$\begin{aligned} S({\varvec{{I}}})_\mu =\frac{1}{m\times n}\sum _{i=1}^{m}\sum _{j=1}^{n}| S({\varvec{{I}}})_{i,j}| \end{aligned}$$

We used a variance threshold of 350 to sort any $S({\varvec{{I}}})_{var}<350$ as blurry. To determine the threshold, we used a simple grid-search scheme from 0 to 525 values incrementing in steps of 175. We inspected the resulting partitions qualitatively as this method required manually determining the threshold value. As a final measure, we qualitatively examined the unfocused X-rays to search potential outliers; we found 35 potential outliers for KL01 and 5 for KL234 , all of which were inserted back into the focused sets. Finally, we manually detected and removed 38 images with surgical prosthetics or X-ray distortions, such as scratches or punch holes. After the focus selection and artifact removal procedure, the final KL01 set contained 3205 images. In comparison , the KL234 set contained 2351 images Fig. 9 showcases four samples, two below and two above the selected Laplace variance threshold. The rejected X-rays were stored and used in the anonymization and augmentation experiments.

### Generative adversarial neural networks

**Figure 7:**
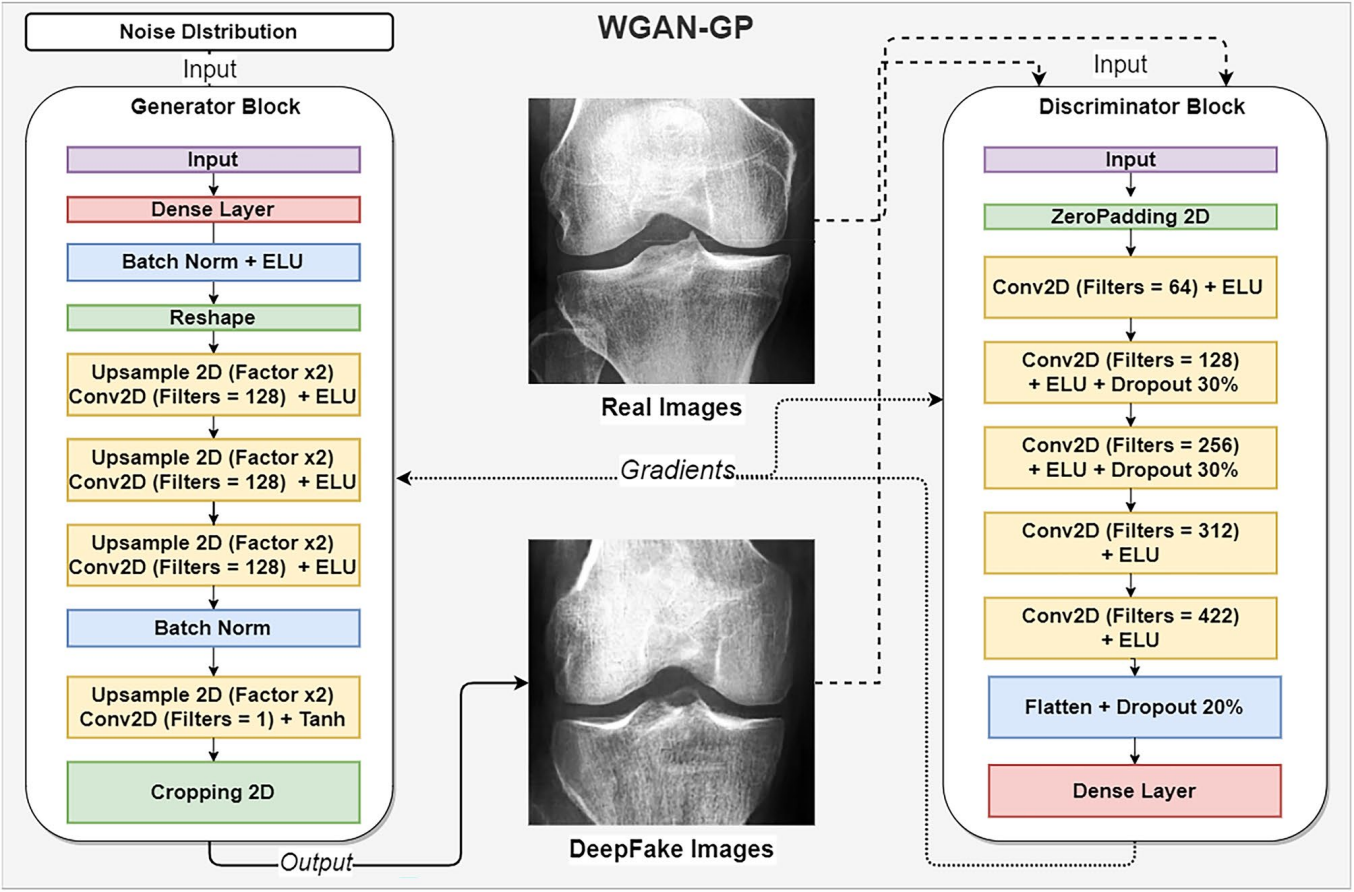
Architecture of the Wasserstein GAN used in these experiments.

We trained two unconditional Wasserstein generative adversarial convolutional neural networks^57^ with gradient penalty^72^. One network instance was trained separately for each combined KL class. The architecture is visualized in Fig. 7 and completely detailed in Table 7. In the original generative neural network formulation^44^, we find two central components, the discriminator *D*(*x*) and the generator *G*(*z*). The two play an adversarial minimax game; the generator tries to deceive the discriminator with counterfeit data samples. The discriminator tries to learn to recognize between real and counterfeit data samples. The GAN minimax objective is defined as (Eq. 5):

5 $$\begin{aligned} J_{gan}(x,\hat{x})={\mathbb {E}}_{x \sim P_{real}} [\log (D(x))] +{\mathbb {E}}_{\hat{x}\sim P_{fake}}[\log (1-D(\hat{x}))] \end{aligned}$$

where *x* are data samples from the data distribution $P_{real}$ and $\hat{x}$ are DeepFake- counterfeit data samples from the data distribution$P_{fake}$ as generated by the generator *G*(*z*) where *z* is sampled from a noise distribution ${Z}_{ \sim n(z)}$. The *D*(*x*) and $D(\hat{x})$ stand for the discriminator’s probability estimate of real images being real, and for DeepFake images being real. GANs formulated in this way suffer from multiple fallbacks, such as the gradient vanishing problem and regular mode collapse. The Wasserstein GAN was later introduced to address some of these issues and remains a widely accepted alternative to the original GAN formulation. The name Wasserstein comes from incorporating the “Earth mover” distance metric, also called Wasserstein-1 cost^73^. This function determines the minimum cost for transforming one distribution into another as the product of mass and distance. In the context of GANs, the Wasserstein-GAN min-max formulation^72^ is as follows (Eq. 6):

6 $$\begin{aligned} J_{wgan}(x,\hat{x})={\mathbb {E}}_{x \sim P_{real}} [(D(x))] -{\mathbb {E}}_{\hat{x}\sim P_{fake}}[(D(\hat{x}))] \end{aligned}$$

**Table 7:** WGAN architecture with intermediate layer shapes and specifications.

Generator architecture	Discriminator architecture
**Layer type—layer parameters(length/shape)**	**Layer type—layer parameters(length/shape)**
Input(50)	Input ($210 \times 210 \times 1$)
Dense—use bias = False(100,352)	Zero padding 2D $2 \times 2$ ($214 \times 214 \times 1$)
Batch normalization (100,352)	Convolution 2D—64 Filters, $5 \times 5$ Kernel, $2 \times 2$ stride ($107 \times 107 \times 64$)
ELU—Alpha = 0.2 (100,352)	ELU—Alpha = 0.2 ($107 \times 107 \times 64$)
Reshape ($14 \times 14 \times 512$)	Convolution 2D—128 filters, $5 \times 5$ kernel, $2 \times 2$ stride ($54 \times 54 \times 128$)
UpSampling 2D—Factor = $\times2$ ($28 \times 28 \times 512$)	ELU—Alpha = 0.2 ($54 \times 54 \times 128$)
Convolution 2D—128 filters, $3 \times 3$ kernel, $1 \times 1$ stride, padding = ‘same’, use bias = False ($28 \times 28 \times 128$)	Dropout—rate: 0.3 ($54 \times 54 \times 128$)
ELU—Alpha = 0.2 ($28 \times 28 \times 128$)	Convolution 2D—256 filters, $5 \times 5$ kernel, $2 \times 2$ stride ($27 \times 27 \times 256$)
UpSampling 2D—Factor = $\times2$ ($56 \times 56 \times 128$)	ELU—Alpha = 0.2 ($27 \times 27 \times 256$)
Convolution 2D—128 Filters, $3 \times 3$ kernel, $1 \times 1$ stride, padding = ‘same’, use bias = False ($56 \times 56 \times 128$)	Dropout—Rate: 0.3 ($27 \times 27 \times 256$)
ELU—Alpha = 0.2 ($56 \times 56 \times 128$)	Convolution 2D—312 filters, $5 \times 5$ kernel, $2 \times 2$ stride ($14 \times 14 \times 312$)
UpSampling 2D—Factor = $\times2$ ($112 \times 112 \times 128$)	ELU—Alpha = 0.2 ($14 \times 14 \times 312$)
Convolution 2D—128 filters, $3 \times 3$ kernel, $1 \times 1$ stride, padding = ‘same’, use bias = False ($112 \times 112 \times 128$)	Convolution 2D—422 filters, $5 \times 5$ kernel, $2 \times 2$ stride ($7 \times 7 \times 422$)
ELU—Alpha = 0.2 ($112 \times 112 \times 128$)	ELU—Alpha = 0.2 ($7 \times 7 \times 422$)
UpSampling 2D—Factor = $\times2$ ($224 \times 224 \times 128$)	Flatten (20,678)
Convolution 2D—1 Filters, $3 \times 3$ kernel, $1 \times 1$ stride, padding = ‘same’, use bias = False ($224 \times 224 \times 1$)	Dropout—Rate: 0.2 (20,678)
Batch normalization ($224 \times 224 \times 1$)	Dense (1)
Tanh ($224 \times 224 \times 1$)	
Cropping 2D—$7 \times 7$ ($210 \times 210 \times 1$)	
Total parameters = 6,304,900	Total parameters = 6,335,861
Trainable parameters = 6,104,194	Trainable parameters = 6,335,861

The two formulations (Eqs. 5, 6) use similar abstractions except that in the latter $D\in {\mathscr {D}}$, where ${\mathscr {D}}$ is a set of 1-Lipschitz functions and is therefore much easier to differentiate. In this instance, *D* no longer outputs a binary classification response as either real or DeepFake, but a numeric result. The *D* trains to learn a 1-Lipschitz continuous function, which in turn assists in computing the Wasserstein distance. In WGAN terminology, the new discriminator is called a critic; as the numeric output of *D* grows smaller (or larger if inverted), the distance between $P_{real}$ and $P_{fake}$becomes smaller. To enforce 1-Lipschitz functions, the original W-GAN used the weight clipping technique that limits the minimum and maximum weights between values $[-c,c]$. This regularization approach was shown to underperform against the gradient penalty^72^ approach that we used. The definition in terms of the loss where $\lambda$ controls the extent of the penalty to the gradients $\Vert \nabla _{\hat{x}}D(\hat{x}) \Vert {_2}$ is shown in Eq. (7):

7 $$\begin{aligned} J_{wgan-gp}(x,\hat{x})={\mathbb {E}}_{\hat{x}\sim P_{fake}}[(D(\hat{x}))] - [(D(x))] + \lambda {\mathbb {E}}_{x \sim P_{fake}}[(\Vert \nabla _{\hat{x}}D(\hat{x}) \Vert {_2}-1)^2] \end{aligned}$$

#### Experiment architecture and parameters

The generator input was a noise distribution *z* , randomly sampling 50 values from the standard normal distribution. The gradient penalty was set to $\lambda =10$, with the discriminator training three extra steps ahead of the generator. Our WGAN architectures for *D* and *G* used convolutional neural networks with activations of the exponential linear unit ( “ELU^74^”) and hyperbolic tangent ( “Tanh”); Our architecture was based on the WGAN-GP model found in the official Keras^75^ repository. Detailed specifications are shown in Table 7. Both the discriminator and the generator trained with the Adam optimizer^76^ with parameters $l=0. 0002$, $\beta _1=0. 5$, $\beta _2=0. 9$, $d=1e-4$ , where *l* was the learning rate, *d* was the decay rate, and $\beta _1$ and $\beta _2$ were the decay rates for the first and second moment estimates , respectively. We trained each WGAN for $t=1000$ epochs, with a batch size of 32. We iterated for 1000 epochs independently for KL01 and KL234 , and the total training time was approximately two days. For ease of communication, we referred to the WGAN critic as the discriminator.

#### Validation

**Figure 8:**
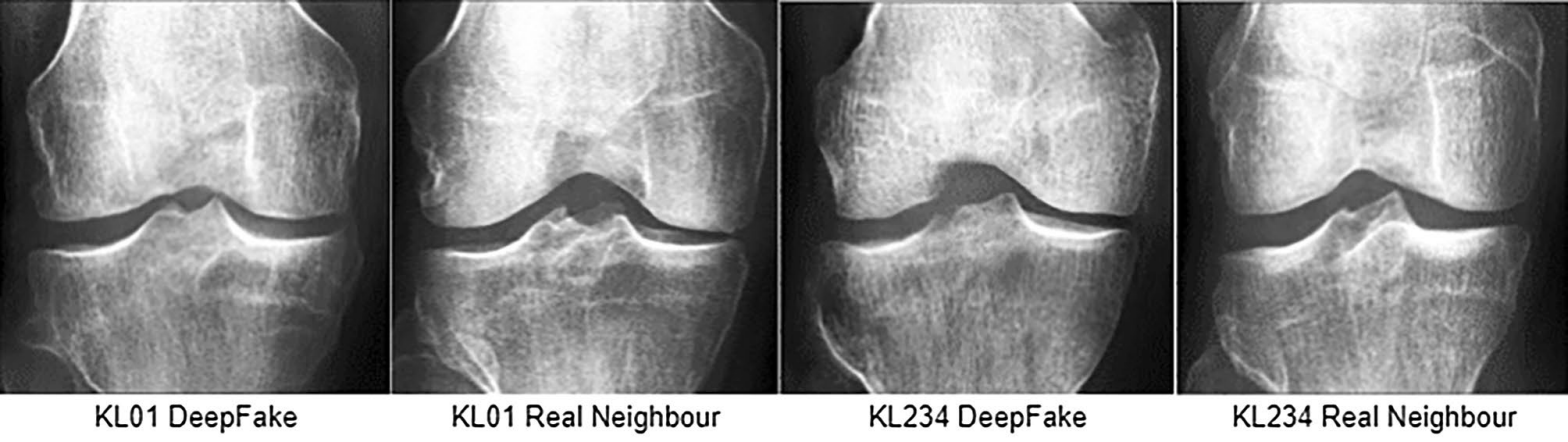
Topmost closest K-nearest neighbors of real images to DeepFake images. The nearest neighbors were computed in InceptionV3 vector space. The first two images are the top closest (shortest distance) KL01 pair, while the second pair are the top closest KL234 pair. These and the remaining sets were shown to the orthopedic surgeon for overfitting validation.

GAN validation was divided into three parts . The first part evaluated WGAN epochs in terms of the Fréchet inception distance (FID)^60^. What followed was an overfitting check with an orthopedic surgeon (model selection). In the second part, medical doctors validated the realism of the selected model. The third part validated DeepFake images ( from the selected models ) for anonymization and augmentation in a KL classification task. We used FID for each epoch model against the real data and obtained a quality metric for the generated images. We measured the generative model sample distribution closest to the real data sample distribution. FID was calculated using features from the InceptionV3^77^ architecture pre-trained with the ImageNet^78^ dataset. Formally, we have a generative model data distribution $P_{model}$ and real data distribution $P_{real}$ . We draw *n* samples from the model distribution $g_1,\ldots ,g_n \sim P_{model}$ and *m* samples from the ‘ ‘real” data distribution $r_1,\ldots ,r_m \sim P_{real}$. The data samples are encoded (feature extraction) with activations as $A(g_i)$ and $A(r_i)$ from the final layer of ImageNetV3 pre-trained inception architecture neural network. Using these activations, the FID is calculated as (Eq. 8):

8 $$\begin{aligned} d_{FID}(A(g_i),A(r_i))=\Vert \mu _g - \mu _r \Vert ^2_2+tr\left( \Sigma _g\right) +tr\left( \Sigma _r\right) -2\cdot tr\left( \sqrt{\Sigma _g+\Sigma _r}\right) \end{aligned}$$

where $\mu _g, \mu _r$ are the corresponding DeepFake and real sample means, *tr* is the trace of the matrix and $\Sigma _g$, $\Sigma _r$ are the covariance matrices of activations $A(g_i)$ and $A(r_i)$. Equation (3) is essentially the Wasserstein distance between multivariate distributions $N(\mu _g,\Sigma _g)$ and $N(\mu _r,\Sigma _r)$. FID was evaluated at each epoch with random generator examples matching the total number of real images ( 3205 for KL01 and 2351 for KL234 ). Minimum FID was found at epoch 647 for the KL01 WGAN and epoch 730 for the KL234 WGAN. We furthered validation with K-nearest neighbors (KNN) between DeepFake images and all real images used for training. The DeepFake images were randomly generated to match the count of real images. KNN was performed in the InceptionV3 vector space (pre-trained with ImageNet) . We sorted all image feature pairs (DeepFake, real neighbors) by their Euclidean distances to one another. We presented the top 20 image pairs of each class to a collaborating orthopedic surgeon. We asked the surgeon to identify if: a) image pairs shared identical or partly identical morphological and clinical features; (b) if the image pairs had any similarities that indicated a common origin. Both cases investigated potential overfitting. The orthopedic surgeon’s evaluation was negative for all image pairs. Thus, we continued with these models as the final selection. Examples of this approach from the top two pairs of each model (KL01, KL234) can be seen in Fig. 8. The entire set evaluated is available in the data availability statement link.

### Medical expert validation

After model selection, we devised a survey to identify images as real or DeepFake. The rationale was to investigate the degree of realism of DeepFake images. We randomly generated 15 KL01 and 15 KL234 images. We randomly obtained the equivalent number of real images, for a total of 30 real and 30 DeepFake images. All survey images were randomly selected with the “random” Python module. The module ran without any explicitly seeded state to avoid potential interference or biases. The images were added to the survey in randomized order and re-scaled to 315 $\times$ 315 pixels. In addition, we asked the medical experts to rate all survey images in terms of KL grades. We distributed this survey to 10 radiologists and 5 orthopedic surgeons, all experts in osteoarthritis diagnostics in the central Finland healthcare district. The survey had 16 respondents; one was disqualified due to their medical specialization in dentistry. Three experts did not provide ratings for some of the images: one in 3 images, another in 1 image, and the last in 20 images. The KL rating section had 12 respondents, two of whom had only 1 rating missing. We dealt with imbalanced responses by using the balanced accuracy metric^79^. The metric is shown below (Eq. 9):

9 $$\begin{aligned} BalAcc = \frac{1}{2}\left( \frac{TP}{TP+FN}+\frac{TN}{TN+FP}\right) \end{aligned}$$

where *TP* are true positives, *FN* are false negatives, *TN* are true negatives and *FP* are false positives. The expression is the equivalent of average recall in each class. The metric allowed obtaining an accuracy with class-balanced sample weights; when two class weights were equal, the expression became exactly equivalent to standard accuracy. When the class weights were unequal, the true class prevalence ration weighted each sample.

### Anonymization and classification augmentation

We investigated the anonymization and augmentation potential of the selected models in a data-scarce scenario. In this setting we devised a transfer learning experiment to classify between the merged classes KL01 and KL234. We used a simple variant of the VGG16^64^ architecture (Table 6) pre-trained with ImageNet, further trained for 22 epochs, with only the last three blocks of the architecture trainable and all remaining blocks frozen. We created six datasets ; we began with real data and progressively added more DeepFake data to create each dataset. The initial dataset represented a typical data-scarce scenario and contained 464 real images divided into three sets. The training set contained 200 images (100 per class), while the testing and validation sets contained 132 images each (66 per class). The augmentation datasets were constructed upon the initial dataset, for which training data increased with DeepFake images by +50%, +100%, +150%, and +200%. The images increased recursively, so the previous set was the starting point for the next augmented set. Finally, the real data were replaced entirely with the DeepFake data for the anonymization experiment. The replaced data were equivalent to removing the real data from the 100% augmentation set. The total number of images in each set is given in Table 5. DeepFake images were generated randomly. Real images were randomly selected with the “random” module from the Python language. To avoid potential selection biases, the module was not explicitly random state-seeded.

**Figure 9:**
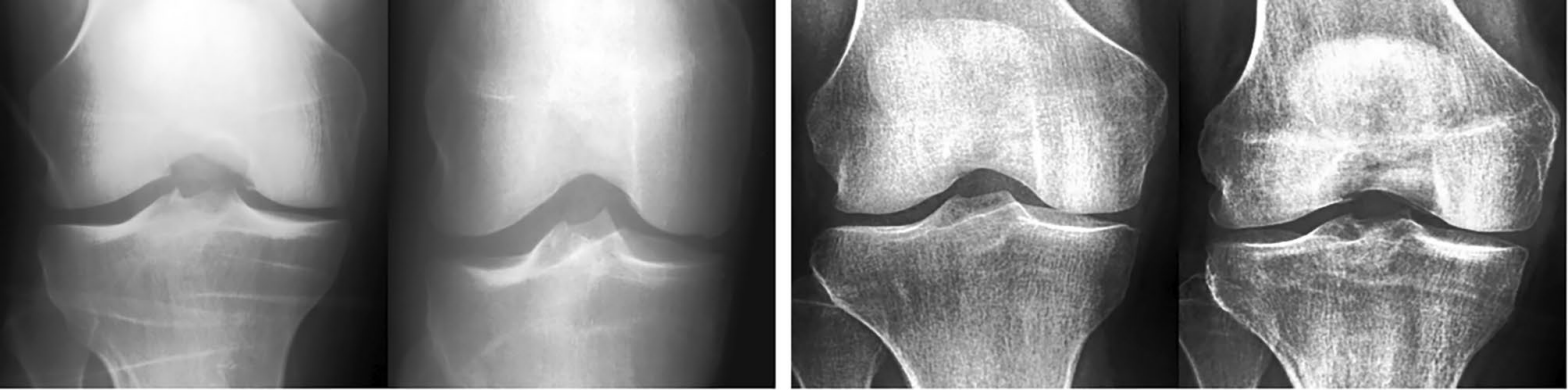
The first two images from the left are below the focus threshold , and the remaining two are above it.

## Data Availability

The datasets generated and/or analysed during the current study are available from Mendeley Data at https://data.mendeley.com/datasets/fyybnjkw7v .
